# Long-Term Effects of an Internet-Mediated Pedometer-Based Walking Program for Chronic Obstructive Pulmonary Disease: Randomized Controlled Trial

**DOI:** 10.2196/jmir.5622

**Published:** 2016-08-08

**Authors:** Marilyn L Moy, Carlos H Martinez, Reema Kadri, Pia Roman, Robert G Holleman, Hyungjin Myra Kim, Huong Q Nguyen, Miriam D Cohen, David E Goodrich, Nicholas D Giardino, Caroline R Richardson

**Affiliations:** ^1^Pulmonary and Critical Care Medicine SectionVA Boston Healthcare SystemBoston, MAUnited States; ^2^Harvard Medical SchoolBoston, MAUnited States; ^3^Pulmonary & Critical Care DivisionUniversity of Michigan Health SystemAnn Arbor, MIUnited States; ^4^Department of Family MedicineUniversity of MichiganAnn Arbor, MIUnited States; ^5^Center for Clinical Management ResearchVA Ann Arbor Healthcare SystemAnn Arbor, MIUnited States; ^6^Department of BiostatisticsSchool of Public HealthUniversity of MichiganAnn Arbor, MIUnited States; ^7^Department of Research and EvaluationKaiser Permanente Southern CaliforniaPasadena, CAUnited States; ^8^Pulmonary Medicine SectionVA New York HarborBrooklyn, NYUnited States; ^9^Department of PsychiatryUniversity of MichiganAnn Arbor, MIUnited States

**Keywords:** bronchitis, chronic, emphysema, pulmonary disease, chronic obstructive, quality of life, exercise, motor activity, Internet

## Abstract

**Background:**

Regular physical activity (PA) is recommended for persons with chronic obstructive pulmonary disease (COPD). Interventions that promote PA and sustain long-term adherence to PA are needed.

**Objective:**

We examined the effects of an Internet-mediated, pedometer-based walking intervention, called Taking Healthy Steps, at 12 months.

**Methods:**

Veterans with COPD (N=239) were randomized in a 2:1 ratio to the intervention or wait-list control. During the first 4 months, participants in the intervention group were instructed to wear the pedometer every day, upload daily step counts at least once a week, and were provided access to a website with four key components: individualized goal setting, iterative feedback, educational and motivational content, and an online community forum. The subsequent 8-month maintenance phase was the same except that participants no longer received new educational content. Participants randomized to the wait-list control group were instructed to wear the pedometer, but they did not receive step-count goals or instructions to increase PA. The primary outcome was health-related quality of life (HRQL) assessed by the St George’s Respiratory Questionnaire Total Score (SGRQ-TS); the secondary outcome was daily step count. Linear mixed-effect models assessed the effect of intervention over time. One participant was excluded from the analysis because he was an outlier. Within the intervention group, we assessed pedometer adherence and website engagement by examining percent of days with valid step-count data, number of log-ins to the website each month, use of the online community forum, and responses to a structured survey.

**Results:**

Participants were 93.7% male (223/238) with a mean age of 67 (SD 9) years. At 12 months, there were no significant between-group differences in SGRQ-TS or daily step count. Between-group difference in daily step count was maximal and statistically significant at month 4 (*P*<.001), but approached zero in months 8-12. Within the intervention group, mean 76.7% (SD 29.5) of 366 days had valid step-count data, which decreased over the months of study (*P*<.001). Mean number of log-ins to the website each month also significantly decreased over the months of study (*P*<.001). The online community forum was used at least once during the study by 83.8% (129/154) of participants. Responses to questions assessing participants’ goal commitment and intervention engagement were not significantly different at 12 months compared to 4 months.

**Conclusions:**

An Internet-mediated, pedometer-based PA intervention, although efficacious at 4 months, does not maintain improvements in HRQL and daily step counts at 12 months. Waning pedometer adherence and website engagement by the intervention group were observed. Future efforts should focus on improving features of PA interventions to promote long-term behavior change and sustain engagement in PA.

**ClinicalTrial:**

Clinicaltrials.gov NCT01102777; https://clinicaltrials.gov/ct2/show/NCT01102777 (Archived by WebCite at http://www.webcitation.org/6iyNP9KUC)

## Introduction

Physical activity (PA) is significantly reduced in persons with chronic obstructive pulmonary disease (COPD), even at the earliest stages of disease [1-3]. Its clinical course is punctuated with acute exacerbations, during and following which persons suffer further reductions in PA [4,5]. As a disease with systemic consequences, COPD increases vulnerability to frailty, immobility, and loss of functional independence. Despite optimal pharmacological therapy, persons with COPD suffer from a downward spiral of breathlessness, deconditioning, and physical inactivity [6]. Comorbidities of cardiovascular disease, diabetes mellitus, and osteoporosis contribute to further reductions in PA [7,8].

Physical activity is a modifiable health behavior that affects COPD-specific outcomes [9-14]. It has been shown that a greater quantity of low-intensity PA reduces risk of COPD hospitalizations, whereas high-intensity PA does not result in risk reduction [15]. In a cohort of persons with COPD, those who walk the least have risks that are 2 and 6 times higher for acute exacerbations and COPD-related hospitalizations, respectively, compared to those who walk the most [12]. In addition, persons with COPD with higher PA levels have a significantly lower risk of dying, independent of forced expiratory volume in 1 second (FEV_1_) [14]. The Global Initiative for Chronic Obstructive Lung Disease (GOLD) guidelines recommend regular PA for all persons with stable COPD as part of standard nonpharmacological treatment [6].

Despite the evidence and recommendations, effective long-term PA interventions are lacking in the clinical care of patients with COPD. Most studies of long-term exercise interventions have examined methods to maintain exercise in the subset of persons with COPD who have completed a conventional pulmonary rehabilitation program [16-21]. These interventions have combined weekly- or monthly-supervised exercise classes with unsupervised home exercise, support groups, and/or telephone contact with a health care professional, showing mixed results over the long term [16-21]. Strategies that promote behavior change and long-term adherence to effectively sustain PA in all persons with COPD are needed.

We developed an automated, Internet-mediated, pedometer-based walking program called Taking Healthy Steps to promote PA in persons with COPD. Taking Healthy Steps combines the Omron HJ-720 ITC pedometer (Omron Healthcare, Inc, Bannockburn, IL, USA) with a disease-specific website accessed via a URL. Taking Healthy Steps provides iterative step-count feedback, individualized step-count goals, education on disease self-management, motivational support, and an online community of social support [22-27]. We studied the efficacy of Taking Healthy Steps in a randomized controlled trial (trial registration: Clinicaltrials.gov NCT01102777) [27]. The conceptual framework, study design, and results at 4 months have been described previously [26,27]. We have shown that Taking Healthy Steps is safe and engaging, and improves health-related quality of life (HRQL) and increases daily step count at 4 months [25-27]. In this study, our primary aim was to assess the long-term efficacy of Taking Healthy Steps on HRQL and daily step counts, a marker for walking behavior change, at 12 months. Our secondary aim was to assess long-term engagement with the PA intervention.

## Methods

### Recruitment

The study design and methods have been reported previously [26,27]. Participants were enrolled from national patient care databases of US Veterans, between December 2011 and January 2013, who had received any treatment services in the previous year and had a COPD diagnosis. Zip codes were matched with the Rural Urban Commuting Area Codes to determine whether one’s residence was urban or rural [28]. Of the 21 regional Veteran Integrated Service Networks (VISN) across the 50 United States and Puerto Rico, we excluded Veterans from one VISN (VISN-1) where another COPD research study using the Taking Healthy Steps platform was recruiting participants. The coordinating center was located at the Ann Arbor VA Healthcare System, Ann Arbor, MI, USA. Ethical approval for this study was granted by the VA Ann Arbor Healthcare System Human Studies Subcommittee.

**Figure 1:**
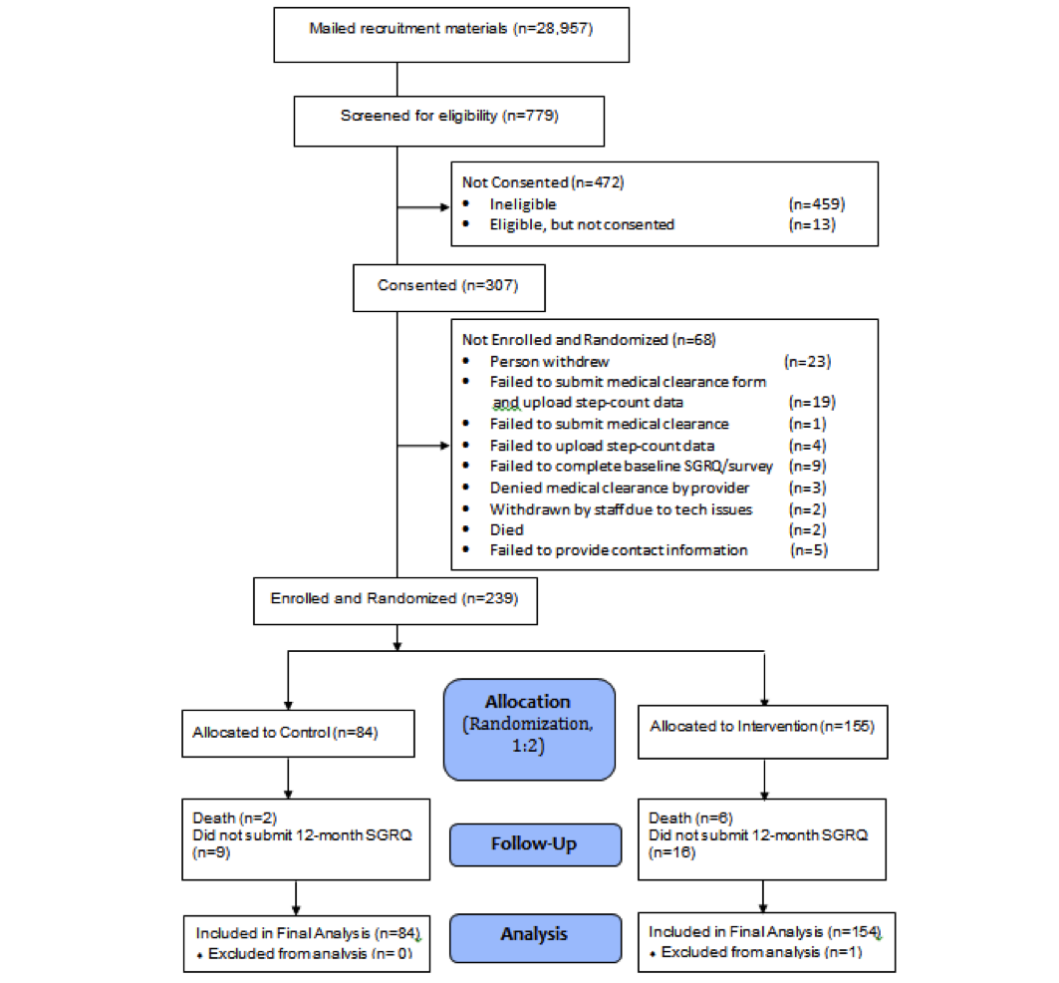
CONSORT diagram at 12 months.

A random sample of 28,957 Veterans (half rural, half urban) with a COPD diagnosis was sent a recruitment letter. Inclusion criteria included having access to a computer with an Internet connection, a USB port, and Windows XP, Vista, Windows 7, or Windows 8. Our a priori exclusion criteria excluded those who did not upload baseline step-count data or who did not complete the baseline survey to assess HRQL. Per study protocol, participants had to have baseline values for the primary (HRQL) and secondary outcome (daily step count) to be enrolled and randomized. Ultimately, 239 participants were enrolled and randomized in a 2:1 ratio to either Taking Healthy Steps (Internet-mediated, pedometer-based walking program) or wait-list control (pedometer alone), stratified by Modified Medical Research Council (MMRC) dyspnea score and urban versus rural status (Figure 1). All participants were prompted monthly to report new or worsening medical problems; all self-reported adverse events were recorded. There were no face-to-face encounters with staff; all features were automatically delivered via the website.

### Outcomes

#### Primary Outcome

The St. George’s Respiratory Questionnaire (SGRQ), a disease-specific instrument with 50 items that has been well validated in COPD [29,30] was used to assess HRQL. It has a summary total score (SGRQ-TS) composed of three domain scores: symptoms (frequency and severity), activities (that cause or are limited by breathlessness), and impact (social functioning and psychological disturbances resulting from airways disease). Scores range from 0 to 100 with lower scores indicating better HRQL. A change of four units is the minimum clinically important difference for the SGRQ-TS [31]. Study participants completed the SGRQ online at study entry, 4 months, and 12 months.

#### Secondary Outcome

Daily step count was assessed by the Omron HJ-720 ITC pedometer. Once participants completed the baseline survey, study staff mailed them a pedometer that had an embedded USB port, an upload cable, and detailed written instructions on how to install the Java software and upload pedometer data. For users who did not have Java already installed on their computers, the software installation was a one-time event. Thereafter, participants uploaded step-count data using the cable that connected the pedometer to their home computer. Research staff were available by telephone to assist with software installation and upload of step counts.

A wear day with valid step-count data was defined as one having at least 100 steps and 8 hours of step counts recorded [32]. At baseline, participants wore the pedometer covered with a sticker to blind the participant to device feedback. Baseline daily step count was the mean daily step count calculated using at least 5 days of valid data within a period of seven consecutive days. Follow-up daily step counts were calculated within a window of +/-14 days around day 121 for 4-month values, and +/-14 days around day 366 for 12-month values. Follow-up daily step counts were the means of at least 5 days of valid data within a period of seven consecutive days. We also calculated the mean daily step count each month by examining the data in 30-day increments. We used values from the last valid week (at least 5 days of valid data within a period of seven consecutive days) in each of those months.

### Intervention Group

Participants randomized to Taking Healthy Steps completed an intensive 4-month intervention period, followed by a distinct 8-month maintenance phase (Table 1). During the first 4 months, participants were instructed to wear the pedometer every day, reminded to upload at least weekly, and were provided access to the website. The website has four key components [26,27]: individualized goal setting was based on uploaded step counts, iterative feedback allowed self-monitoring of step counts, motivational content provided a new educational tip every other day and a new motivational message each week, and an online community forum enhanced social support [22-27]. During the 8-month maintenance phase, participants continued to wear the pedometer, upload daily step counts, receive weekly step-count goals and feedback, and had access to the online community forum. They could view the initial 4 months of educational content and motivational messages, but no longer received new content. Topics on the online community forum included walking in a variety of weather/seasons, health topics (weight management, COPD disease management), injury prevention, barriers to walking, and technical issues with the pedometer and website.

**Table 1:** Features available to the Taking Healthy Steps and control groups during the first 4 months versus last 8 months of the study.

Features	0-4 Months		5-12 Months	
	Taking Healthy Steps	Control	Taking Healthy Steps	Control
Wear pedometer	Yes	Yes	Yes	Yes
Upload step-count data	At least weekly	At least monthly	At least weekly	At least monthly
Goal setting	Yes	No	Yes	No
Feedback	Yes	No	Yes	No
New educational and motivational content	Yes	No	No	No
Online community forum	Yes	No	Yes	No

### Wait-List Control Group

Participants randomized to the wait-list control group were instructed to wear the pedometer every day, reminded monthly to log in to the website to upload step-count data, and asked to report all adverse events. Veterans in the wait-list control group received neither instruction to increase PA nor step-count goals. They had access to a webpage that showed only a checklist of surveys completed and a count of what week they were in the study. After 12 months, they were given the option to use the Internet-mediated intervention.

### Participant Characteristics, Device Adherence, and Website Engagement

At baseline, participants answered questions online that assessed comorbidities, oxygen use, smoking status, and demographics. At study entry, 4 months, and 12 months, dyspnea was assessed using the MMRC scale (range 0-4 with 4 indicating the most severe level of dyspnea) [33]. Events self-reported during the study were defined a priori as COPD-related if persons experienced a combination of symptoms and/or required treatment with antibiotics and/or systemic corticosteroids. The COPD-related events included acute exacerbations or pneumonia, ascertained by self-reported events and/or review of health care utilization (hospitalizations and emergency room visits) and pharmacy data. To assure independence of individual acute exacerbations, participants were considered to have experienced a new acute exacerbation only if it were reported 21 or more days after the previous acute exacerbation [34].

We examined device adherence, overall and by group, by calculating the percentage of days (of 366 days) that were wear days with valid step-count data. For the participants who uploaded valid step-count data at 12 months and completed the study, we also examined percentage of days (of 42 days) that were wear days during the last 6 weeks of the study.

In the intervention group, we objectively examined website engagement by recording the number of log-ins to the website by month of study and assessing the frequency of use of the online community forum. In addition, at 4 and 12 months, participants in the Taking Healthy Steps group answered a structured survey eliciting feedback about their commitment to their step-count goals and various aspects of engagement with the intervention, including participants’ ease of finding time to log in to the website, knowledge of step-count goals, and use of the different components of the website.

### Statistical Analysis

Proportions, means, and standard deviations described baseline participant characteristics. Two-sample *t* tests and chi-square tests compared baseline characteristics between groups. The occurrence of COPD-related events (acute exacerbations or pneumonia), hospitalizations, emergency room visits, deaths, and adverse events during the study were each compared between groups using a logistic regression model. For the count of hospitalizations, a zero-inflated Poisson regression model was also used to assess the difference in the rate of hospitalizations between groups. These models adjusted for age, gender, treatment group, and oxygen use.

The primary analysis used the intention-to-treat approach, and used a linear mixed-effects model with baseline, 4-month, and 12-month outcome values (eg, SGRQ-TS or daily step count) as dependent variables. No baseline variable was predictive of missingness in models adjusting for stratification variables and treatment group. Thus, the longitudinal data model included participants who had the dependent variable for at least one time point and was expected to give unbiased estimates of the intervention effect assuming missingness at random. The model included participants as random intercepts to adjust for within-participant correlations of repeated measures, fixed predictors of treatment group, 4- and 12-month time indicators, and treatment group by time indicator interactions, MMRC dyspnea score (dichotomized to 0-1 vs 2-4), and urban versus rural residence. We also analyzed the data excluding those who died. The proportion of participants who had at least a 4-unit improvement in SGRQ-TS at 12 months was compared between groups using a chi-square test [31]. For the analysis of mean daily step count by month of study, we used a linear mixed-effect model similar to that for the primary outcome except data were assessed in 30-day increments over the 12-month study period. Predictors were treatment group, month of study as indicator variables (coded as 1-12), group-by-month indicator variables interactions, dichotomized MMRC dyspnea score, and urban versus rural residence.

We assessed website engagement in the intervention group by characterizing the number of log-ins to the website using the mean, median, and interquartile range, and assessed trends over months of study using a linear mixed-effects regression analysis with monthly number of log-ins for each participant as the outcome and time (month since randomization) as the predictor. Trends for device adherence over month of study were examined with percent of days with valid step-count data using a linear mixed-effects model and for use of the online community forum using a generalized mixed-effects model with logit link. The effect of time on participant responses to the online survey about goal commitment and intervention engagement at 4 and 12 months was estimated for each response variable using a mixed-effects model with 4- and 12-month survey data as the dependent variable and predictors including 12-month indicator, baseline dichotomized MMRC dyspnea score, and urban versus rural status. All models, including the model for the number of log-ins, were checked for model assumptions using residuals.

One participant in the Taking Healthy Steps group was considered an outlier given that his change in SGRQ-TS was 4.0 standard deviations greater than the mean for change in SGRQ-TS and his change in daily step count was 8.1 standard deviations greater than the mean for change in daily step count. The extremely high step counts more likely reflected his occupational PA rather than any effects of our intervention. Our main analyses excluded the outlying individual, but we also repeated primary and secondary outcome analyses with this participant included. All analyses were performed with Stata 14.0 (StataCorp LP, College Station, TX, USA).

## Results

### Participant Characteristics

No information is available on the persons to whom we mailed recruitment materials but who were not screened because they did not go to our website and did not call us (Figure 1). The top three reasons for ineligibility of 459 participants were not sedentary (n=202), could not walk a block (n=120), or no compatible computer access (n=161), with some participants having more than one reason (Figure 1). In all, 68 persons consented but were not enrolled and randomized, including 19 who failed to submit a medical clearance form and did not upload step-count data, one who failed to submit a medical clearance form, four who failed to upload step-count data, and nine who failed to complete the baseline SGRQ (Figure 1).

Participants’ (N=238) characteristics include: mean age 67 (SD 9) years, male (93.7%, 223/238), rural residence (45.4%, 108/238), MMRC dyspnea score ≥2 (30.7%, 73/238), current smokers (24.8%, 59/238), and supplemental oxygen use (23.5%, 56/238) (Table 2). There were no significant differences in baseline characteristics between study groups, including current smoking history. Overall, 87.8% (209/238) of participants completed the 12-month online HRQL assessment, and 74.4% (177/238) uploaded 12-month valid step-count data. In the intervention group, 87.7% (135/154) of participants completed the HRQL assessment and 76.6% (118/154) uploaded valid step-count data, compared to 88% (74/84) and 70% (59/84), respectively, in the control group.

**Table 2:** Baseline participant characteristics (N=238).

Characteristic		Intervention (n=154)	Control (n=84)	Total (N=238)
Age (years), mean (SD)		67 (8.6)	66.4 (9.2)	66.8 (8.8)
Gender (male), n(%)		146 (94.8)	77 (92)	223 (93.7)
**Residence, n(%)**				
	Urban	83 (53.9)	47 (56)	130 (54.6)
	Rural	71 (46.1)	37 (44)	108 (45.4)
Hispanic (n=235), n(%)		5 (3.3)	1 (1)	6 (2.6)
**Race, n(%)**				
	Black	7 (4.6)	3 (4)	10 (4.2)
	White	142 (92.2)	79 (94)	221 (92.9)
	Other	5 (3.3)	2 (2)	7 (2.9)
Current smoker, n(%)		41 (26.6)	18 (21)	59 (24.8)
Oxygen use, n(%)		35 (22.7)	21 (25)	56 (23.5)
**SGRQ,^a^ mean (SD)**				
	Symptoms	57.2 (19.1)	56 (19.9)	56.8 (19.3)
	Activities	62.3 (20.2)	64.2 (18)	62.9 (19.5)
	Impact	32.2 (16.5)	34.1 (17.9)	32.9 (17)
	Total	45.6 (15.4)	46.8 (15.6)	46 (15.4)
Baseline daily step count, mean (SD)		3488 (2316)	3521 (2058)	3499 (2224)
**MMRC dyspnea score,^b^ n (%)**				
	0-1	108 (70.1)	57 (68)	165 (69.3)
	2-4	46 (29.9)	27 (32)	73 (30.7)

^a^SGRQ: St. George’s Respiratory Questionnaire. Data for symptoms, activities, and impact were available from 236 participants; total from 233 participants.

^b^MMRC: Modified Medical Research Council.

At 12 months, 29 of 238 (12.2%) participants did not have sufficient data to calculate the SGRQ-TS: 19 Taking Healthy Steps participants and 10 controls. There was no significant difference in baseline SGRQ-TS (mean 49.8, SD 16.1 vs mean 45.6, SD 15.3; *P=*.18) or baseline daily step count (mean 3410, SD 2667 vs mean 3512, SD 2163; *P=*.82) between those for whom SGRQ-TS could not be calculated (n=29) versus those for whom SGRQ-TS was calculated at 12 months (n=209).

The percent of participants with COPD-related events (acute exacerbations or pneumonia) during the study did not differ between groups (control: 18%, 15/84; intervention: 22.7%, 35/154; logistic regression OR 1.4, 95% CI 0.7-2.8; *P=*.33). No between-group difference was found in the percent of participants with hospitalizations (control: 17%, 14/84; intervention: 23.4%, 36/154; logistic regression OR 1.6, 95% CI 0.8-3.2; *P=*.19) or emergency room visits (control: 24%, 20/84; intervention: 29.9%, 46/154; logistic regression OR 1.4, 95% CI 0.8-2.6; *P=*.27) during the 12-month study. For the count of hospitalizations, a zero-inflated Poisson regression model also found no between-group difference. The percent of participants who died during the study did not differ between groups (control: 2%, 2/84; intervention: 3.9%, 6/154; *P=*.53). Finding no between-group differences in the percentage of participants who were hospitalized or died provided assurance that the censoring of the outcome variables (SGRQ-TS or daily step counts) due to these events was not likely to confound the assessment of the between-group outcome differences. However, we repeated the analyses with deaths excluded as well.

### Health-Related Quality of Life

There was no significant between-group difference in the primary outcome of SGRQ-TS (mean 1.1 units, 95% CI –2.2 to 4.5; *P=*.50) at 12 months (Table 3). The proportion of participants who achieved at least a 4-unit improvement in SGRQ-TS at 12 months was 45.2% (61/135) in the intervention versus 32% (23/71) in the control group (*P=*.08). There was no significant between-group difference in the SGRQ domain scores of symptoms (mean 0.5 unit, 95% CI –4.2 to 5.2; *P=*.84), activities (mean 0.04 unit, 95% CI –4.2 to 4.2; *P=*.99), and impact (mean 2.3 units, 95% CI –1.6 to 6.1; *P=*.25) at 12 months.

**Table 3:** Within-group changes and between-group differences in SGRQ scores and daily step counts at 12 months.

Outcome and arm			N	Difference from baseline to 12 months, mean (95% CI)	*P*	Between-group difference, mean (95% CI)	*P*^a^
**SGRQ**							
	**Total**					1.1 (–2.2, 4.5)	.50
		Taking Healthy Steps	154	–2.5 (–4.5, –0.6)	.01		
		Control	84	–1.4 (–4.1, 1.3)	.31		
	**Symptoms**					0.5 (–4.2, 5.2)	.84
		Taking Healthy Steps	154	–3.2 (–6.0, –0.4)	.02		
		Control	84	–2.7 (–6.5, 1.1)	.16		
	**Activities**					0.04 (–4.2, 4.2)	.99
		Taking Healthy Steps	154	–1.2 (–3.7, 1.3)	.36		
		Control	84	–1.1 (–4.5, 2.3)	.51		
	**Impact**					2.3 (–1.6, 6.1)	.25
		Taking Healthy Steps	154	–3.4 (–5.6, –1.1)	.004		
		Control	84	–1.1 (–4.2, 2.0)	.48		
**Daily step count**						–108 (–720, 505)	.73
	Taking Healthy Steps		154	270 (–86, 626)	.14		
	Control		84	163 (–336, 661)	.52		

^a^ Based on linear mixed-effect models, adjusting for group, 4- and 12-month indicators, group×time indicator interactions, baseline MMRC dyspnea score (dichotomized to 0-1 vs 2-4), and urban versus rural status.

Intervention participants showed an improvement in SGRQ-TS of a mean 2.5 units (95% CI –4.5 to –0.6) at 12 months, compared to baseline (*P=*.01) (Table 3). For domain scores in the intervention group, symptoms improved by a mean 3.2 units (95% CI –6.0 to –0.4, *P=*.02), and impact improved by a mean 3.4 units (95% CI –5.6 to –1.1, *P=*.004) at 12 months. The control group showed no significant changes in the SGRQ-TS and domain scores at 12 months compared to baseline (Table 3). When the analysis was repeated with the outlying individual included, no substantive difference was seen in results, except improvement in symptoms within the Taking Healthy Steps group was marginally significant (*P*=.05). When the analysis excluded the eight deaths, results remained nearly identical.

### Daily Step Count

**Figure 2:**
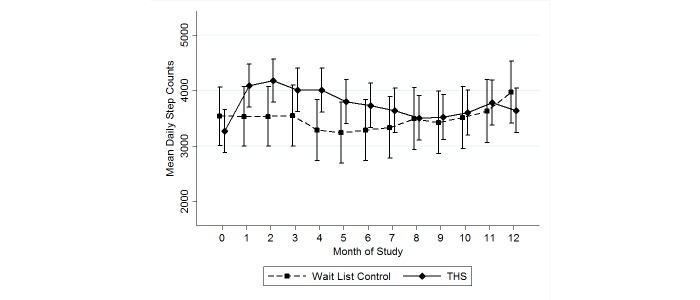
Mean daily step count and 95% confidence intervals by month of study. Note: the Taking Healthy Steps (THS) curve is shifted to the right of the control curve on the x-axis for ease of visual display. Baseline data are included at month zero.

There was no significant difference between groups with respect to the secondary outcome of daily step count at 12 months (*P=*.73) (Table 3). There was no significant change in daily step count in the intervention participants (*P=*.14) or in the control group (*P=*.52) at 12 months, compared to baseline (Table 3). Examination of daily step count by month of intervention showed that differences in daily step counts in the intervention group compared to controls were maximal and statistically significant at month 4, but approached zero in months 8 to 12 (Figure 2). Between-group *P* values were <.001 at 4 months, .28 at 8 months, and .82 at 12 months. Within the intervention group, although daily step counts peaked at 2 months and then declined over the course of the study, daily step counts continued to be higher than baseline values in all months of the study (Figure 2). Analysis including the outlying individual showed improvement in daily step counts at 12 months to be significant in the Taking Healthy Steps group (*P*=.048). Analysis excluding the eight deaths did not change results.

### Device Adherence and Website Engagement

**Figure 3:**
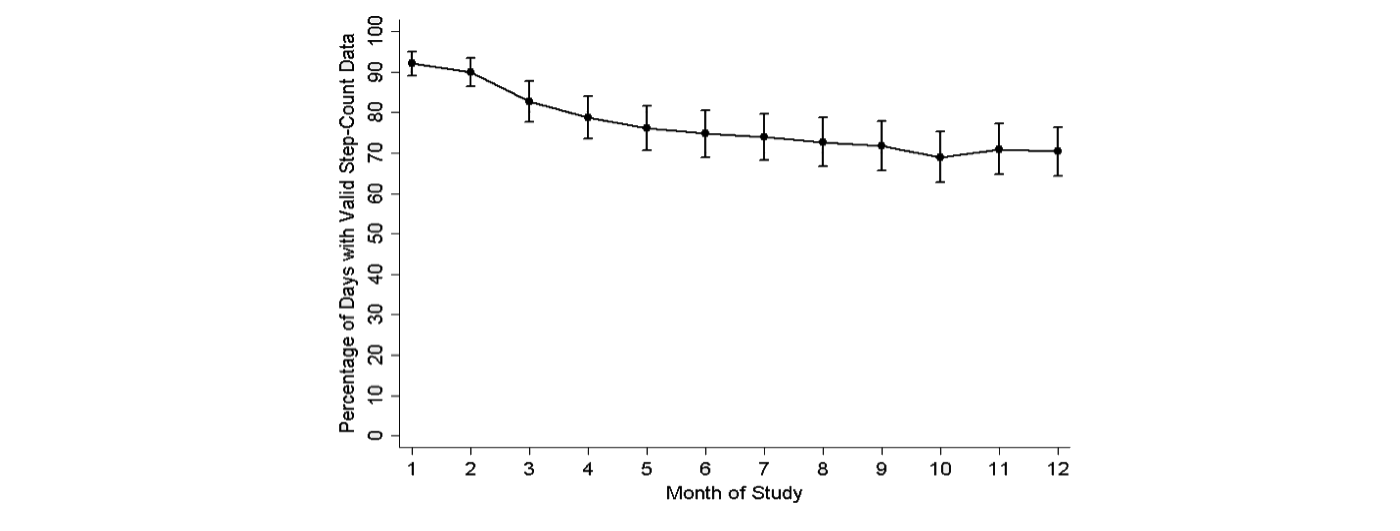
Percentage and 95% confidence intervals of days with valid step-count data in the intervention group by month of study.

Device adherence during the 12-month study period was significantly higher in the intervention group than the control group, with mean 76.7% (SD 29.5) of the 366 days having valid step-count data in the intervention group versus mean 63.7% (SD 32.9) of the 366 days having valid step-count data in the control group (*P*=.002). For the 177 participants in both groups who uploaded valid step-count data at 12 months and completed the study, mean 83.1% (SD 21.9) of days in the last 6 weeks of the study had valid step-count data. In these last 6 weeks, mean 87.5% (SD 16.5) of days had valid step-count data in the intervention group, which was significantly higher than the mean 74.1% (SD 28.1) of days observed in the control group (*P*<.001). In the intervention group, device adherence decreased significantly over time (*P*<.001), with mean 92.1% (95% CI 86.6-97.6) of days having valid step-count data at month 1 versus 70.3% (95% CI 64.9-75.8) of days at month 12 (Figure 3).

**Figure 4:**
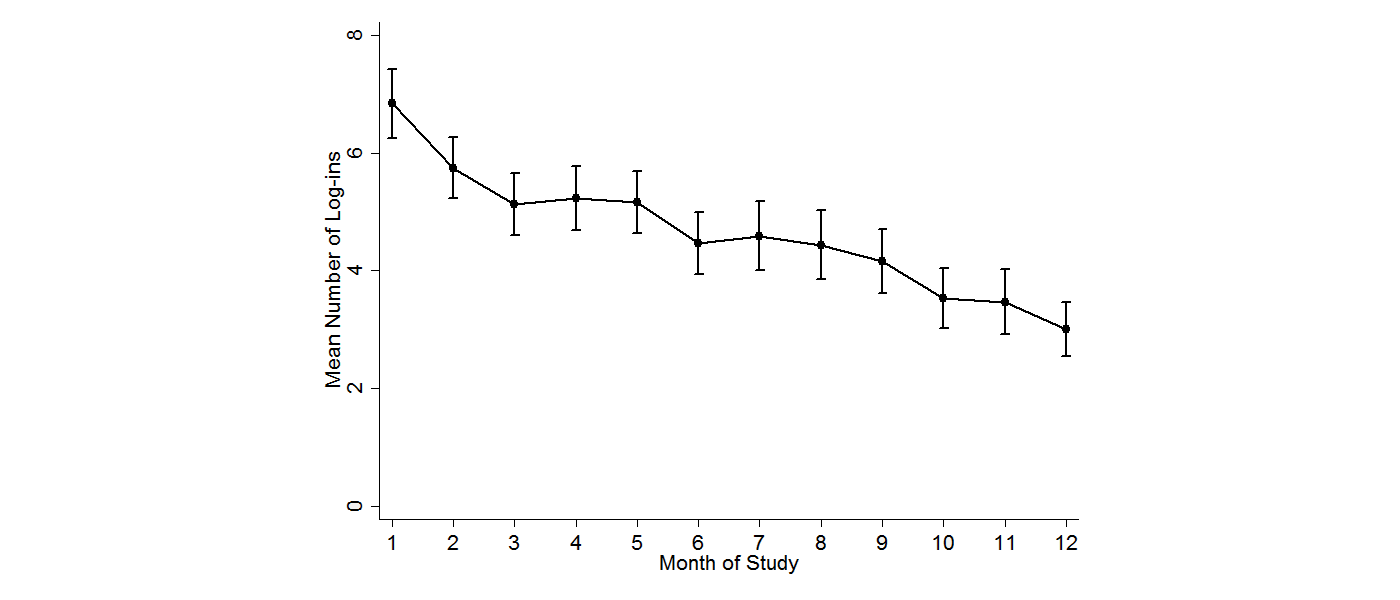
Mean log-ins and 95% confidence intervals in the intervention group by month of study.

In the intervention group, mean number of log-ins to the website decreased significantly over the months of study (*P*<.001; Figure 4). The number of monthly log-ins was mean 6.8 (SD 3.7; median 6, IQR 3) at month 1, which declined to mean 4.2 (SD 3.5; median 4, IQR 3) by month 9 and mean 3.0 (SD 3.0; median 3, IQR 5) by month 12 (Figure 4). In the intervention group, 83.8% (129/154) of the participants used the online community forum at some point during the 12-month study; 66.2% (102/154) of participants directly viewed an online community forum thread or entry, and an additional 17.5% (27/154) of participants posted a new topic or a reply at least once. More than half of the participants responded “definitely true” (22/121, 18.2%) or “mostly true” (45/121, 37.2%) to the statement: “I learned helpful information when I used the online community forum.” There was a significant trend for decreasing use of the online community forum by month of study (*P*<.001).

Responses to questions regarding participant’s goal commitment were not significantly different at 12 months compared to 4 months (Table 4). When asked, “Overall, how motivated are you to walk each day?” with responses from 1=not motivated and 10=extremely motivated, the mean response was 6.8 (SD 2.3) at 4 months compared to mean 6.5 (SD 2.5) at 12 months (*P=*.06). Responses to questions about engagement with the use of Taking Healthy Steps were not significantly different at 12 months compared to 4 months (Table 4).

**Table 4:** Goal commitment and engagement with Taking Healthy Steps intervention.

Goal commitment and engagement		N^a^	4 months mean (95% CI)	12 months mean (95% CI)	*P*^b^
**Goal commitment^c^**					
	It’s hard to take my step-count goal seriously.	147	2.1 (1.9-2.2)	2.0 (1.9-2.2)	.69
	Quite frankly, I don’t care if I reach my step goal or not.	147	1.7 (1.6-1.8)	1.7 (1.6-1.8)	.46
	I am strongly committed to pursuing my step-count goal.	146	3.8 (3.6-4.0)	3.7 (3.5-3.9)	.52
	It wouldn’t take much to make me abandon my step-count goal.	147	1.9 (1.7-2.1)	2.0 (1.8-2.2)	.27
	I think my step-count goal is a good goal to shoot for.	146	4.0 (3.8-4.2)	3.9 (3.8-4.1)	.77
**Engagement in Taking Healthy Steps^d^**					
	I would recommend the Taking Healthy Steps walking program to another person with COPD.	146	1.3 (1.2-1.4)	1.2 (1.1-1.3)	.01
	It was easy for me to find the time to log in to the website once a week.	146	1.8 (1.6-2.0)	1.8 (1.6-2.0)	.92
	I had technical difficulty uploading step-count data from the pedometer to my computer.	146	4.0 (3.7-4.2)	3.9 (3.7-4.1)	.75
	I knew what my step goal should be every day.	147	1.5 (1.4-1.6)	1.5 (1.4-1.6)	.48
	I was able to comfortably increase my daily step count every week.	147	2.6 (2.5-2.8)	2.8 (2.6-3.0)	.10
	I looked at the graphs of the step counts that I walked.	147	1.6 (1.4-1.7)	1.6 (1.4-1.7)	.76
	The motivational messages and educational tips were easy to understand.	143	1.9 (1.8-2.0)	1.8 (1.7-1.9)	.21
	I learned helpful information when I used the online community forum.	137	2.5 (2.3-2.7)	2.4 (2.3-2.6)	.52
	The daily step-count goals were too high for me to walk each day.	147	3.4 (3.2-3.5)	3.4 (3.2-3.5)	.98

^a^ Participants with responses at 4 and/or 12 months were included in the models.

^b^ Based on linear mixed-effect models with 4 and 12 months as the dependent variable and predictors of 12-month indicator, intervention group indicator and baseline MMRC dyspnea score (dichotomized to 0-1 vs 2-4) and urban versus rural status.

^c^ Response scale 1-5 with 1=strongly disagree, 2=disagree, 3=neither agree nor disagree, 4=agree, 5=strongly agree.

^d^ Response scale 1-5 with 1=definitely true, 2=mostly true, 3=not sure, 4=mostly false, 5=definitely false.

### Safety

Adverse events were categorized as pulmonary, cardiac, musculoskeletal, or other. A significantly greater percent of participants in the intervention group (27.9%, 43/154) had minor musculoskeletal adverse events than in the control group (10%, 8/84; *P*<.001). There were no differences between groups with respect to pulmonary, cardiac, or other adverse events during the 12 months.

## Discussion

We show that our Internet-mediated, pedometer-based walking intervention does not maintain benefits in HRQL and daily step counts at 12 months, despite demonstrated improvements at 4 months [27]. Although we report negative findings for the study overall, lessons learned about device adherence and website engagement are highly informative for guiding the development of future PA interventions that can effectively promote long-term behavior change and sustain PA.

Overall, we found that a COPD population found the study feasible and were engaged. The percentage of participants who completed the 12-month study, providing HRQL and step-count data, was high. In addition, our objective results assessing device adherence and showing that 83% of days for participants in both groups within the last 6 weeks of the study had valid step-count data support that people were not lost to the study and then showing up for the last evaluation period. Importantly, persons in the intervention group had significantly higher device adherence compared to the control group for the study overall and at the end of the study. This finding supports that the goal setting, feedback, educational and motivational content, and online community forum provided on the website significantly increased intervention engagement beyond that observed with the use of a pedometer alone.

For the first time, we rigorously elicited participant survey responses about goal commitment and intervention engagement, and objectively assessed device adherence and website engagement during the 12-month study. In the intervention group, responses to questions about engagement at 12 months were the same compared to those at 4 months, with participants finding time to log in to the intervention, knowing their step-count goal, and using the graphs, tips and messages, and forums. They also reported that they were as committed to their step-count goal at 12 months as they were at 4 months. Although participants reported the same levels of goal commitment and intervention engagement at the end of the study compared to the beginning of the study, sustained behavior change was not observed because there were significant decreases in number of days with valid step-count data, number of log-ins to the website, and use of the online community forum over time. Although we can only speculate as to cause and effect, the decrease in daily step count (a marker of intervention efficacy and walking behavior change) over time mirrors the declines in device adherence and website engagement over time.

The reasons for the observed decline in daily step counts over time require further exploration. Participants may not have continued to wear the pedometer, log in to the website, and walk over the 12 months for a variety of possible reasons that we did not assess, such as waning interest with the intervention, progression of underlying COPD, flare-up of comorbidities, or occurrence of intercurrent life events (eg, spouse illness). The effect of the intervention on daily step counts could potentially have been greater if the control group had not received a pedometer and monthly reminders to upload step counts. We are confident that battery life did not affect the results because we mailed a new battery with replacement instructions to each participant every 4 months. We replaced lost or broken devices reported to us.

These results are similar to published data examining maintenance exercise programs after conventional pulmonary rehabilitation [16-21]. Typically, the unstable clinical course of a chronic lung disease such as COPD makes it difficult for patients to resume or maintain an exercise program [20]. Although we observed no difference in the number of COPD-related events, such as acute exacerbations, between groups, the occurrence of acute exacerbations and flare-up of comorbidities over a period of 12 months may have modified the response to Taking Healthy Steps within the intervention group.

The failure to obtain long-term benefits with our PA interventions parallels the literature studying other behavior changes, such as smoking cessation [35] and weight loss [36]. Our 8-month maintenance phase retained the key components of goal setting, feedback, and social support. The main feature omitted beginning at month 5 was new educational and motivational content. These findings support that ongoing behavioral modification techniques are critical to sustain PA [37,38]. We speculate that additional intervention components, such as face-to-face contact with peers and/or health care providers, would enhance the social support and motivation needed to sustain PA as a routine behavior. Use of evolving technology, such as wireless transmission and mobile connectivity with cell phones, smartphones/mobile phones, or tablets, could potentially provide anytime/anywhere access to the PA intervention and enhance its long-term efficacy [37,39,40]. Intensive counseling and support at the time of acute exacerbations and flare-up of comorbidities would address medical barriers to PA and motivate patients to continue to walk after an illness. Finally, incorporating the health care provider, health care institutions, communities, and society at large into PA interventions could enhance long-term behavior change and adherence to effectively sustain PA in persons with COPD [41,42].

The exact role of digital walking programs in starting and maintaining exercise in persons with COPD remains to be determined. Both acute and chronic models of digital walking programs are potentially useful. Acute intervention models are needed to initiate and promote PA in the vast majority of patients with COPD who cannot access a conventional pulmonary rehabilitation program [43]. In addition, maintenance models are appropriate and much needed because long-term maintenance of behavior change is challenging. In addition, digital walking programs can potentially be useful adjuncts after conventional pulmonary rehabilitation to maintain benefits, which start to wane as early as 3 to 6 months after program completion [20,21]. They can also be an important component of COPD self-management programs [44]. An interesting future question to address is whether restarting our intervention every 4 to 8 months would be an efficacious long-term strategy.

The potential full impact of our intervention can only be appreciated by performing a future cost-effectiveness analysis. Results from cross-sectional data from our group and others have shown that every step counts. We have not found a “threshold” or “optimal” daily step count to obtain clinical benefits. The benefits appear to be linear such that those with higher step counts have lower risks for acute exacerbations, hospitalizations, hospital admissions and readmissions, and death compared to those with lower step counts [9-14]. Future work is needed to examine whether PA interventions such as ours can decrease health care resource utilization and result in cost savings to our health care system.

Major strengths of our study include the randomized controlled trial design with balanced groups at baseline, objective data on device adherence and website engagement, and the long-term follow-up of 12 months. Our intervention is based on a theoretical model, and informed by previous work eliciting patient feedback to optimize user acceptability and develop the motivational and educational content [25,45]. Our Internet-mediated, pedometer-based intervention focuses specifically on walking, a low-intensity PA that most patients can do. It has already been shown that a greater quantity of low-intensity PA reduces risk of COPD hospitalizations, whereas high-intensity PA does not result in risk reduction [15].

Our study has several limitations. We studied primarily white male Veterans limiting the generalizability of our results. Spirometric confirmation of the COPD diagnosis was not made at study entry. However, any potential misclassification of asthma as COPD was most likely balanced between groups and would not bias the primary results. The vast majority of the patients had MMRC <2. It is justified to include patients with MMRC <2 because patients with newly diagnosed COPD have reduced PA even at the earliest stages of the disease [3]. It is important to promote PA even when patients are not significantly symptomatic, as recommended by the GOLD guidelines for COPD [6]. We found no difference in benefit of the PA intervention in those with MMRC <2 versus MMRC ≥2. We acknowledge the final response rate was likely biased toward responders who had a particular interest in this type of intervention, and the results may not be generalizable to a wider COPD population. Finally, seasonal variation can influence our secondary outcome of daily step counts. We minimized the impact of season by having a 12-month intervention and enrolling participants over all four seasons.

An Internet-mediated, pedometer-based PA intervention for persons with COPD does not maintain improvements in HRQL or daily step count at 12 months, despite demonstrated improvements at 4 months. In addition, waning engagement with the PA intervention support that future efforts should focus on improving features of PA interventions to enhance long-term behavior change and sustain engagement with PA. These findings need to be considered when designing future Internet-mediated PA interventions.

## Multimedia Appendix 1

CONSORT-EHEALTH checklist.

## Abbreviations

AE: acute exacerbations
COPD: chronic obstructive pulmonary disease
FEV^1^: forced expiratory volume in 1 second
GOLD: Global Initiative for Chronic Obstructive Lung Disease
HRQL: health-related quality of life
MMRC: Modified Medical Research Council
PA: physical activity
SGRQ: St George’s Respiratory Questionnaire
SGRQ-TS: St George’s Respiratory Questionnaire Total Score
VISN: Veteran Integrated Service Network
